# Role of carnitine in disease

**DOI:** 10.1186/1743-7075-7-30

**Published:** 2010-04-16

**Authors:** Judith L Flanagan, Peter A Simmons, Joseph Vehige, Mark DP Willcox, Qian Garrett

**Affiliations:** 1 Institute for Eye Research, Sydney, New South Wales, Australia; 2 Allergan Inc, Irvine, CA, USA; 3 School of Optometry and Vision Science, University of New South Wales, Sydney, Australia

## Abstract

Carnitine is a conditionally essential nutrient that plays a vital role in energy production and fatty acid metabolism. Vegetarians possess a greater bioavailability than meat eaters. Distinct deficiencies arise either from genetic mutation of carnitine transporters or in association with other disorders such as liver or kidney disease. Carnitine deficiency occurs in aberrations of carnitine regulation in disorders such as diabetes, sepsis, cardiomyopathy, malnutrition, cirrhosis, endocrine disorders and with aging. Nutritional supplementation of L-carnitine, the biologically active form of carnitine, is ameliorative for uremic patients, and can improve nerve conduction, neuropathic pain and immune function in diabetes patients while it is life-saving for patients suffering primary carnitine deficiency. Clinical application of carnitine holds much promise in a range of neural disorders such as Alzheimer's disease, hepatic encephalopathy and other painful neuropathies. Topical application in dry eye offers osmoprotection and modulates immune and inflammatory responses. Carnitine has been recognized as a nutritional supplement in cardiovascular disease and there is increasing evidence that carnitine supplementation may be beneficial in treating obesity, improving glucose intolerance and total energy expenditure.

## Introduction

Carnitine (β-hydroxy-γ-*N*-trimethylaminobutyric acid) is widely distributed in food from animals sources but there is limited availability in plants [1]. In humans, 75% of carnitine is obtained from the diet [2]. L-carnitine (the biologically active stereoisomer) is absorbed from foods via both active and passive transport across enterocyte (intestinal cell) membranes [3]. The bioavailability of L-carnitine varies due to dietary composition. Bioavailability of L-carnitine in individuals such as vegetarians who are adapted to low-carnitine diets is higher (66% to 86% of available carnitine) than regular red-meat eaters adapted to high-carnitine diets (54% to 72% of available carnitine) [4]. Carnitine not obtained from food is synthesized endogenously from two essential amino acids, lysine and methionine. This occurs in kidney, liver and brain [5]. Cardiac and skeletal muscle, harboring the highest concentrations, cannot synthesize carnitine and so must acquire carnitine from plasma. Unabsorbed L-carnitine is mostly degraded by microorganisms in the large intestine [3]. Almost all carnitine (99%) is intracellular [5]. Carnitine influences carbohydrate metabolism. Aberrations in carnitine regulation are implicated in complications of diabetes mellitus, hemodialysis, trauma, malnutrition, cardiomyopathy, obesity, fasting, drug interactions, endocrine imbalances and other disorders.

The purpose of this review is to summarize the role of carnitine in human nutrition and disease and highlight the major areas of research in this field.

### Carnitine biosynthesis and metabolism

**Figure 1:**
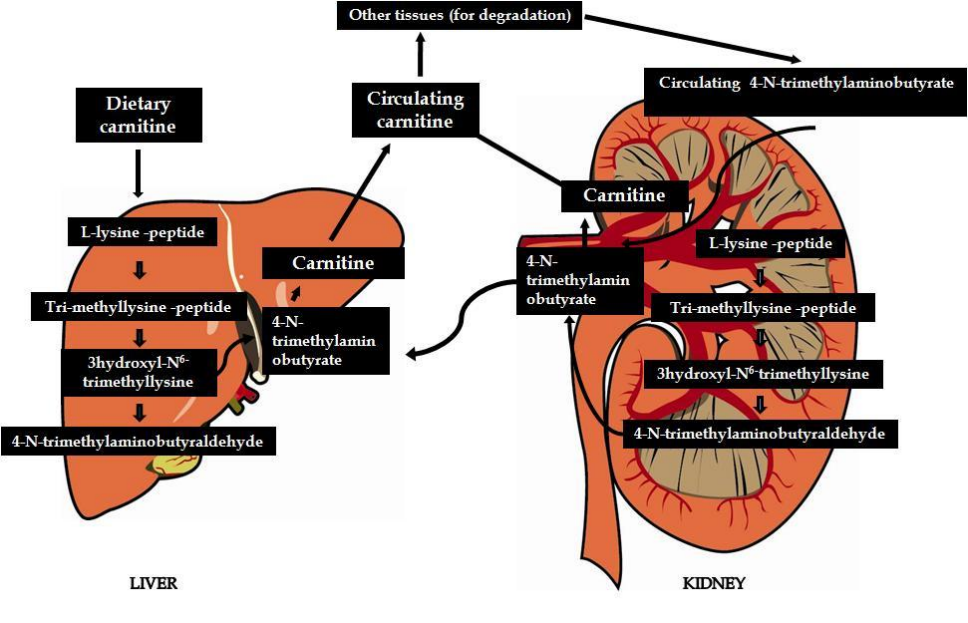
**Carnitine biosynthesis and metabolism**.

Carnitine, a branched non-essential amino acid, is synthesized from the essential amino acids lysine and methionine. Ascorbic acid, ferrous iron, pyroxidine and niacin are also necessary cofactors [1] and deficiencies of any of these can lead to carnitine deficiency. The pathway in mammals is unique using protein-bound lysine that is enzymatically methylated to form trimethyllysine as a post-translational modification of protein synthesis [6]. Trimethyllysine undergoes four enzymatic reactions in the course of endogenous L-carnitine biosynthesis (Figure 1).

One of the enzymes in this pathway, γ-butyrobetaine hydroxylase, is absent from cardiac and skeletal muscle but highly expressed in human liver, testes, and kidney [4]. The rate of L-carnitine biosynthesis in vegetarians is estimated to be around 1.2 μmol/kg of body weight/day [7]. Omnivorous humans ingest 2-12 μmol/kg of body weight/day which represents 75% of body carnitine sources [8]. Neither renal reabsorption nor changes in dietary carnitine intake appear to affect the rate of endogenous carnitine synthesis [9]. Bioavailability of oral carnitine dietary supplements is only in the order of 14 to 18% of dose and unabsorbed L-carnitine is mostly degraded by micoorganisims in the large intestine [3].

**Figure 2:**
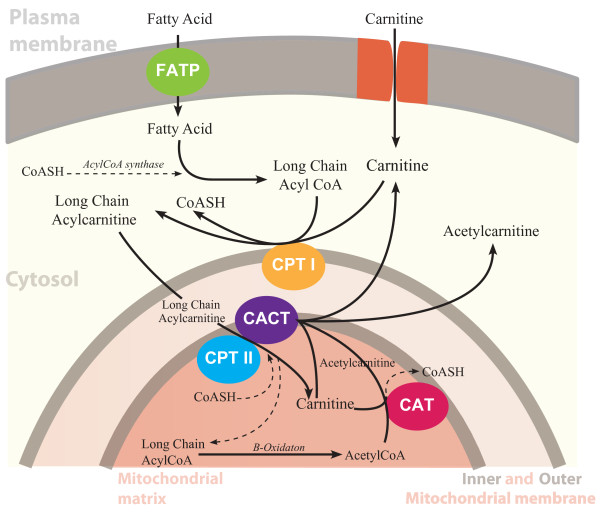
**Carnitine is actively transported via OCTN2 into the cytosol to participate in the shuttling of activated long chain fatty acids into the mitochondria where β-oxidation takes place**. Carnitine also regulates the Coenzyme A (CoA)/acylCoA ratio within the mitochondria, modulation of which reduces accumulation of toxic acyl-CoA compounds and maintains energy production.

Free L-carnitine, absorbed from dietary intake or synthesized in liver and kidney, reaches the blood stream and the extracellular fluid. Its transport within cells of various tissues is limited by their respective uptake capacities [10]. Plasma concentration of free carnitine is in dynamic balance with acylcarnitines with the acyl to fee carnitine ration of ≤ 0.4 being considered normal [11]. Acetylcarnitine esters are formed intracellularly during regular metabolic activity. Long chain acetylcarnitine esters transport fatty acyl moieties into the mitochondria (Figure 2). Short and medium-chain acetyl esters, formed in the mitochondria and peroxisomes, participate in the removal of organic acids [12]. Acetyl-L-carnitine is the principal acylcarnitine ester [12]. Acetyl-L-carnitine participates in both anabolic and catabolic pathways in cellular metabolism [12].

Carnitine plays a critical role in energy balance across cell membranes and in energy metabolism of tissues that derive much of their energy from fatty acid oxidation such as cardiac and skeletal muscles [13,14] (Figure 2). Although carnitine plays its main role in carnitine free fatty acid metabolism, it also enhances carbohydrate utilization [15]. Uptake in skeletal and cardiac muscle is a saturable active transport process against a concentration gradient [16].

Experimental evidence suggests that the transport of long chain fatty acids into the mitochondria is a rate limiting step in fatty acid oxidation. During sustained low to moderate exercise, fatty acid oxidation increases to become the predominant energy source to muscles [17]. CPTI (Figure 2) is a control point of FA oxidation and decreased carnitine levels and acidosis of CPT1 have been implicated in decreased fatty acid oxidation during heavy exercise [18]. Deficiencies in CPTII may result in exercise induced muscle injury due to inability to increase FA oxidation with increased exertion.

Carnitine participates in cell volume and fluid balancing in all tissues that are affected by the tonicity (iso-, hyper- hypo- tonicity) of the extracellular environment [19]. Data suggest that despite fluctuations in carnitine concentration due to its osmolytic pressure changes, carnitine maintains its energy production capacities and often osmolytic gradients can be harnessed for energy[19]. Carnitine fluctuates with both physiological and pathological changes in osmotic pressure. In one example of a physiological response to osmotic pressure, in early mammary gland milk production osmoregulatory pathways are exploited using asymmetric kinetics to increase the carnitine concentration in milk for suckling neonates who have reduced carnitine stores, even though this results in decreased maternal liver stores [20].

### Primary Carnitine Deficiency Syndromes

Two distinct carnitine deficiency states have been reported although a rigid distinction between "primary" and "secondary" carnitine deficiency is difficult to establish in some cases [10]. Primary carnitine deficiency (PCD) is a rare autosomal recessive disorder of fatty acid oxidation caused by deficiency of plasma membrane carnitine transport resulting from impairment in the plasma membrane OCTN2 carnitine transporter. This deficiency restricts tissue uptake, leading to decreased accumulation in the heart and skeletal muscle and potentiates increased renal carnitine loss [21,22] leading to systemic carnitine depletion [23]. Due to defective renal absorption (free) carnitine is excreted in the urine of patients with primary deficiency and can result in tissue carnitine levels dropping to below 10% of normal [14,22,24-26]. Genetic deficiencies of transporter activity represent the only known forms of primary carnitine deficiency [27].

PCD occurs in 1-5 per 10, 000 population and most commonly manifests between ages 1-7 [28,29]. The most common presentation of PCD is hypoketotic hypoglycemic encephalopathy. Cardiomyopathy has also be observed [30]. The gene responsible for PCD is SLC22A5. Several mutations have been described [21,22,25,26,29,31-33]. Three tissues/organs are affected in PCD: cardiac muscle which leads to progressive cardiomyopathy; central nervous system which is affected by encephalopathy caused by hypoketotic hypoglycaemia, and skeletal muscle which is affected by myopathy [30]. For these patients, L-carnitine supplementation is a life-saving treatment.

Three distinct clinical entities have been described; the adult, the infantile, and the perinatal, all with an autosomal recessive inheritance pattern [34]. The different mutations in SLC22A5 probably give rise to differences in severity/onset of the disease. Measurement of free carnitine and total carnitine in plasma are important in the diagnosis.

Carnitine transporter mutations in Crohn's disease consists of missense mutation(s) in the gene coding plasma membrane transporter OCTN1 (SLC22A4) and/or mutation(s) in the promoter of the gene encoding OCTN2 (SLC22A5) [14,35]. Manifestation of these mutations results in disruption of a heat shock binding element decreasing the transport function (OCTN1), and reduced expression (through OCTN2 mutation) which both result in carnitine deficiency [14]. These mutations are in strong linkage disequilibrium, creating a two-allele risk haplotype and hence increasing the overall risk of this disease [14].

### Secondary Carnitine deficiency

Secondary deficiency is characterized by increased carnitine excretion in urine in the form of acyl-carnitine due to an accumulation of organic acids [36,37]. Secondary carnitine deficiency can be caused by increased losses, pharmacological therapy, a number of inherited metabolic disorders [38], poor diet or malabsorption of carnitine, from increased renal tubular loss of free carnitine (Fanconi syndrome), haemodialysis, peritoneal dialysis, or the increased excretion of acylcarnitines[39] with certain drugs. There have been reported at least 15 syndromes in which carnitine deficiency seems to be secondary to genetic defects of intermediary metabolism or to other conditions [40]. Patients with secondary carnitine deficiency accumulate organic acids which causes enhanced urinary excretion of carnitine in the form of acyl-carnitines [36,37].

Secondary carnitine deficiency (SCD) is less severe with respect to its short-term clinical impact and is much more common [23]. As opposed to PCD, SCD occurs due to, or in association with, other disorders such as liver or kidney disease, defects in fatty acid metabolism, or administration of pharmacological agents such as pivampicillin or valproic acid (discussed below) [27,41,42]. SCD is seen in patients with renal tubular disorders, in which there may be excessive excretion of carnitine, and in hemodialysis patients. A lack of carnitine in dialysis patients is caused by insufficient carnitine synthesis and by the loss through dialytic membranes, leading, in some patients, to carnitine depletion and a concomitant relative increase in esterified forms of carnitine [43]. L-Carnitine supplementation an lead to improvements in several complications seen in uremic patients, including cardiac complications, impaired exercise and functional capacities, muscle symptoms, increased symptomatic intradialytic hypotension, and erythropoietin-resistant anemia through normalizing the reduced carnitine palmitoyl transferase activity in red cells [43]. Argani and colleagues showed a decrease in plasma levels of triglyceride (TG) and very low-density lipoprotein, and increases in total high-density lipoprotein cholesterol (HDL-C and HDL2-C) and albumin, in hemodialysis patients upon administration of 500 mg/day carnitine taken orally for 2 months [44].

### Secondary genetic carnitine deficiency

#### CPT-1 deficiency

Carnitine palmitoyltransferase I (CPTI) deficiency is thought to cause serious disorders of fatty acid metabolism. The nucleotide sequences of cDNA and genomic DNA encoding human CPTI have been characterized [45,46]. However, a relationship between disease and mutation of the human CPTI gene has not been reported [47].

#### CPT II deficiency

The adult CPT II clinical phenotype is somewhat benign and requires additional external triggers such as high-intensity exercise before the predominantly myopathic symptoms are elicited. The perinatal and infantile forms involve multiple organ systems. The perinatal disease is the most severe form and is invariably fatal [34]. The most frequent symptom of muscle palmitoyltransferase CPT II deficiency is an exercise induced myalgia [1]. Myoglobinuria, the traditional hallmark of this disease was not present in 21% patients in one study [48]. Myalgia typically starts in childhood while myoglobinuria starts later in adolescence or early adulthood [1]. One case study found a novel mutation in CPT II (del1737C), an autosomal recessive disease with a distinct phenotype [49]. A two- day old boy died due to severe hepatocardiomuscular disease with an extreme early onset. His sister also died. Upon autopsy the brother showed massive pulmonary atelectasis with intra-alveolar hemorrhage, cardio- and hepato-megaly. The sister died of sudden cardiopulmonary arrest due to the increase of long-chain (C16-18) acylcarnitines. Decreased CPT II activity was found in her liver, heart and kidney. The cause of death was neonatal CPT II deficiency.

### Acquired carnitine deficiency

#### Hemodialysis and kidney disorders

In the kidney, osmolytes including carnitine are crucial since hypertonicity is usual and the kidney must cope with fluctuations of diuresis (increased production of urine) and antidiruesis. Extracellular osmolarity of medullary cells may become more than four-fold that of isotonicity [50]. In healthy individuals, carnitine is freely filtered and tubular resorption of free carnitine (FC) is almost complete. What is excreted in urine is carnitine ester, or acylcarnitine (AC) [13]. In healthy people, the renal clearance of AC is four to eight times that of FC [51-53]. Impairment of excretion of AC occurs with deteriorating renal function leading to decreased carnitine clearance and resulting in elevated plasma levels of carnitine [13]. Uremic patients have elevated levels of AC that occur as both elevated FC and total carnitine before dialysis [52]. These patients experience accumulation of plasma acylcarnitines, in part due to a decreased renal clearance of esterified carnitine moieties [54]. Due to accumulation of metabolic intermediates, impaired carnitine biosynthesis, reduced protein intake, and increased removal of carnitine through hemodialysis (HD), patients who undergo routine HD usually present with plasma carnitine insufficiency [54]. During dialysis, patients experience subnormal plasma/serum free carnitine concentrations [13] with plasma levels dropping by 60% with a slow replacement from carnitine stores such as from skeletal muscle during the interdialytic period.

Dietary intake also plays an important role in carnitine homeostasis of HD patients since the prevalence of malnutrition ranges from 18% to 75% of these cases [54]. Clinical consequences of such malnutrition can lead to impaired muscle function, decreased wound healing, altered ventilatory response, and abnormal immune function [54]. Repeated hemodialytic treatments can result in depletion of skeletal muscle carnitine stores. Intravenous L-carnitine (LC) following dialysis can replenish the free carnitine removed from the blood and restore muscle carnitine content, alleviating muscle myopathies and impaired exercise capacity [13], as well as ameliorating erythropoietin-resistant anemia, decreased cardiac performance, intradialytic hypotension [54]. Furthermore, LC may positively influence the nutritional status of HD patients by promoting a positive protein balance, and by reducing insulin resistance and chronic inflammation, possibly through an effect on leptin resistance [54]. Handelman however cautions that evidence for effectiveness of carnitine supplements in dialysis suffers from trials limited in subject number and open labeled, and suggests more rigorous testing is needed [55].

### Cyclosporin A induced nephrotoxicity

Cyclosporine (CyA) is used as an immunosuppressive agent following organ transplantation but its use is limited due to its associated nephrotoxicity. Bertelli *et al*. [56] demonstrated that L-propionylcarnitine (L-PC), a propionyl ester of L-carnitine, is able to prevent CyA-induced acute nephrotoxicity, reducing lipid peroxidation in the isolated and perfused rat kidney *in vitro*. *In vivo *studies demonstrate that L-PC was able to significantly lower blood pressure in CyA treated animals and to prevent decrease creatinine clearance that normally results from CyA administration [57]. Origlia and colleagues further demonstrated L-PC-associated reduction in lipid hydroperoxide content and morphological abnormalities associated with chronic CyA administration [57].

### Cirrhosis and liver disorders

Carnitine deficiency has been associated with cirrhosis [5]. L-acyl-carnitine has been suggested as a potent, low-cost, and safe alternative therapy for patients with cirrhosis [58]. Minimal hepatic encephalopathy (MHE) is a serious and common complication that occurs in the majority of cirrhotic patients [59]. Malaguarnera and colleagues treating MHE patients with acetyl-L-carnitine (ALC) exhibited recovery of neuropsychological activities related to attention/concentration, visual scanning and tracking, psychomotor speed and mental flexibility, short-term memory, attention and computing ability, language, orientation ability and cognitive activities [59]. There is a strong correlation between hepatic encephalopathy and abnormal ammonia handling, and ALC has been shown to induce ureagenesis leading to decreased blood and brain ammonia levels [60]. This is supported by other studies that showed a protective effect of L-carnitine against ammonia-evoked encephalopathy in cirrhotic patients, with ALC administration improving neurological symptoms and plasmatic parameters in cirrhotic patients with hepatic coma [60-67].

Carnitine depletion is common in patients hospitalized for advanced cirrhosis and results from three factors; substandard intake of dietary carnitine; substandard intake of lysine and methionine; and loss of capacity to synthesize carnitine from these two amino acids [68]. The most likely reason for incapacity to synthesize carnitine from lysine and methionine is inability to convert γ-butyrobetaine to carnitine [68]. Chronic ingestion of alcohol is known to cause hepatic steatosis [69,70]. Sachan *et al*. [71] demonstrated that exogenous carnitine added to the ethanol diet in an experimental rat model significantly reduced lipid accumulation in livers which were otherwise laden with lipids, suggesting that there is a deficiency of functional carnitine i.e. carnitine which is available for acylation [72]. Supplementation of the diet with lysine can restore carnitine levels, however, there appeared to be impairment of carnitine biosynthesis in ethanol-compromised livers in the rat study [71]. It is known that dietary absorption of amino acids is impaired by ethanol so this could also contribute to carnitine deficiency overall [73]. It appears that reduced plasma and peripheral tissue carnitine levels result from sequestration by ethanol-compromised liver [71]. Sachan and colleagues conclude that dietary carnitine is effective in preventing lipid accumulation that results from ethanol-feeding of rats. Dietary carnitine proved to be an effective hypolipidemic agent. Efficacy was related to degree of hypercarnitinemia which is consistent with a deficiency of functional carnitine biosynthesis in the ethanol fed rats [71].

### Obesity, endocrine disorders and diabetes

Evidence is mounting that carnitine supplementation may be beneficial in obesity [5]. In obese rats manifesting insulin resistance, carnitine supplementation improved glucose tolerance and increased total energy expenditure [5]. Carnitine palmitoyltransferase (CPT)-1 is the rate-limiting step of the fatty acid oxidation pathway and a target for the treatment of obesity. Modulation of CPT-1 may affect energy metabolism and food intake, and research is ongoing into the effects of both stimulation and inhibition of CPT-1 and in relation to obesity management [74].

Pharmacological stimulation of brain carnitine palmitoyl-transferase-1 (CPT-1) was reported to decrease food intake and body weight [75]. A selective CPT-1 stimulator produced long lasting hypophagia (reduced food intake) and persistent weight loss [75]. However, this is in contrast with other studies that found CPT-1 *inhibition *actually stimulated hypophagia [76,77] and weight loss [77]. Thus further work needs to be done to clarify this issue. There is some debate in the literature regarding whether satiety depends on the cytosolic concentration of long-chain fatty acids, with the suggestion that an increased concentration correlates with satiety and decreased feeding and body weight [77-80]. However, Aja and colleagues found no evidence for this hypothesis since in this model CPT-1 should inhibit feeding by increasing cytosolic fatty-acyl CoA levels while they actually showed the initial response of mice to a CPT-1 inhibitor was an increase in appetite [75]. The authors discuss whether CNS injection of the drug versus systemic treatment may play an important role in the overall effect.

The development of type 2 diabetes is accompanied by decreased immune function, the underlying mechanisms of which are unclear. It has been suggested that oxidative damage and mitochondrial dysfunction may play an important role in the immune dysfunction in diabetes [81]. This hypothesis was tested using mitochondrial targeting nutrients in a diabetic rat model. Administration of a combination of mitochondrial targeting nutrients, including carnitine, suggested carnitine may be effective in improving immune function in type 2 diabetes through enhancement of mitochondrial function, decreased oxidative damage, and delayed cell death in the immune organs and blood [81].

Glutaryl-CoA dehydrogenase (GCDH) deficiency is an inborn error of lysine and tryptophan metabolism that results in increased formation and excretion of glutaric acid (GA), 3-hydroxyglutaric acid (3-OH-GA), glutaconic acid and glutarylcarnitine [82]. Secondary carnitine depletion due to increased formation and urinary excretion of glutarylcarnitine is suggested to play an important role in the neuropathogenesis of GCDH deficiency, inducing excitotoxic neuronal damage and mitochondrial dysfunction [83]. GCDH can be controlled nutritionally and supplementation includes L-carnitine to avoid secondary carnitine depletion [84-87].

Hyperthyroid patients exhibit higher urinary carnitine concentrations compared with controls while hypothyroid patients exhibit concomitantly lower levels [88]. However ameliorating thyroid therapies normalizes carnitine levels [1].

Patients with type 2 diabetes (particularly those who are insulin dependent or have disease-related complications) seem to be at increased risk for carnitine deficiency [5]. Diabetic polyneuropathy (DPN) is the most common late complication of diabetes mellitus. Experimental rat models of DPN have identified early metabolic abnormalities affecting nerve conduction velocities and endoneurial blood flow [89]. These abnormalities can lead to perturbed lipid peroxidation and expression of neurotrophic factors which ultimately cause degenerative nerve function. As the structural changes progress, they become increasingly less amendable to metabolic interventions. In both experimental models and human diabetic subjects, there is an initial metabolic phase that is responsive to metabolic corrections [90,91]. As the disease progresses however it becomes increasingly non-responsive to therapeutic interventions [92,93].

Acetyl-L-carnitine (ALC) acts on a number of levels in the treatment of type 1 DPN. Clinical trials of ALC have shown ameliorating effects on nerve conduction slowing, neuropathic pain, axonal degenerative changes and nerve fiber regeneration [89].

### Trauma, sepsis and wound healing

The metabolic process in trauma and sepsis includes greatly accelerated proteolysis and resulting protein loss in skeletal muscle [94]. It is known that sepsis patients have depleted carnitine stores at the cellular level [95]. In the liver, the rate of synthesis of selected proteins (i.e., albumin, transferrin, prealbumin, retinol-binding protein, and fibronectin) is decreased, whereas acute phase protein synthesis is accelerated [94]. Tissues characterized by fast replicating cells also show reduced protein synthesis. Carnitine has been trialed in cases of sepsis and found to retard protein loss without affecting protein metabolism in target tissues [94].

The pathophysiology of bacterial-endotoxin mediated tissue damage may involve the interplay of reduced host carnitine levels and pathogenic requirement of carnitine for growth and survival in the host [95]. The endogenous carnitine pool could be a major determinant of mounting an effective immune and inflammatory response towards invading pathogens [95]. This altered carnitine metabolism has been implicated in the multiple organ failure in subjects with systemic inflammatory response syndrome and toxic shock. Carnitine levels are reduced in patients suffering Gram-negative sepsis and urinary loss of carnitine is proportional to the degree of injury [96]. Prophylactic use of carnitine in such situations has been shown to reduce the endothelial damage caused by lipopolysaccharide (LPS) and TNF-α. It has been further suggested that carnitine deficiency might negatively impact cardiac function which might in turn, further contribute to the outcome of patients suffering sepsis [97-99]. There has even been suggestion that maintenance of normal carnitine levels might inhibit muscle wasting, hepatic lipogenesis, hypertriglycerdemia and decreased fatty acid oxidation that are seen in sepsis [95].

A proportion of infants and children with sepsis progress to cardiac failure as part of multiple system organ failure (hepatic, renal, cardiac, pulmonary) [100]. Eaton *et al*. [100] have suggested that inhibition of myocardial CPT I activity may be a common feature of systemic inflammation, or of inflammation localized to the heart.

A study of plasma and urinary levels of free carnitine and short-chain acyl-carnitines in surgical patients showed that the septic state was associated with increased urinary excretion of free carnitine and lower plasma levels of short-chain acyl-carnitines [101]. The authors suggested that theoretically, carnitine supplementation during total parenteral nutrition might be of benefit in sepsis.

Literature regarding wound healing and carnitine is sparse. In relation to burns and wound healing; one study involving 14 patients with severe burns over eight days showed dramatically increased levels of excreted carnitine [102]. There was a positive relationship between extent of burn and carnitine output [1]. Decreased wound healing exhibited by dialysis patients is most likely a consequence of the malnutrition suffered by these patients. McCarty and Rubin suggest supplementation of micronutrients including carnitine to aid wound healing in diabetics [103].

It has been shown that carnitine has a significant dose-dependent effect in promoting random pattern skin flap survival [104]. However Koybasi and Taner found that although there was a tendency toward faster healing, in a group of experimental rats receiving the drug L-carnitine, there was no significant promotion of secondary wound healing [105].

### Malnutrition, fasting and vegetarianism

Reduced plasma carnitine levels have been noted in malnourished children [106] and adults [107]. Levels generally improve with dietary intervention [1]. Kwashiorkor and marasmus represent clinical forms of protein-energy malnutrition (PEM) [108]. Carnitine levels in children suffering PEM are low but reach normal levels following protein repletion [108]. Malnourished children have low levels of many enzymes and it is likely that cofactors for carnitine could be lacking as well. There is a positive correlation between albumin and plasma carnitine levels in PEM and plasma albumin is a widely used indicator of PEM [108]. A negative correlation between free carnitine and both triglycerides and cholesterol indicates that L-carnitine may be utilized under conditions of augmented lipolysis. There have been varying reports of urinary free-carnitine excretion with PEM as either increasing [108] or decreasing [109]. Finally, incremental growth was seen in 22 of 33 carnitine-administered patients who presented with failure to thrive [110] and this was attributed to the role of carnitine as a muscle growth factor.

Experiments with fasted and calorie-restricted rats showed increased mRNA concentrations of acyl-CoA and CPT-1 in the liver, heart and kidneys compared to control animals due to upregulation of PPARα [111]. These studies demonstrated that fasting upregulates the plasma membrane OCTN2 carnitine transporter in the liver, heart, kidneys and in rats with strong caloric restriction, additionally in skeletal muscle [111]. Fasting or caloric restriction was shown to increase the ratio of free carnitine to acetylcarnitine in most tissues analyzed. The authors suggest that the amount of Acetyl-CoA in the mitochondrion available for esterification of free carnitine was reduced in fasted or energy-restricted animals leading to increased tissue carnitine concentrations while acetylcarnitine levels were reduced [111]. These metabolic adaptations during fasting, that are triggered by PPARα, serve to minimize the use of protein and carbohydrates as fuel to allow survival during long periods of energy deprivation.

Oxidized fat was shown to upregulate PPARα and OCNT2 and lead to reduced rate of weight gain compared to controls, indicating an impairment of the feed conversion ratio [112]. Since there is increased OCTN2 expression in the small intestine in response to oxidized fat, and OCTN2 binds not only carnitine but various drugs, it is suggested by the authors that OCTN2 might be harnessed to improve absorption of various drugs [112].

A study by Karlic and colleagues found that a vegetarian diet has a significant impact on genes regulating essential features of carnitine metabolism [113]. Elevated plasma membrane OCTN2 carnitine transporter expression was observed in vegetarians compensating for lower carnitine levels obtained from the diet. Thus a vegetarian lifestyle has an impact on fat metabolism causing a remarkable stimulation of carnitine uptake [113].

### Neuroprotection and dementia

In the brain, the role of carnitine in isotonicity is crucial since alteration of tonicity would affect nerve excitability due to ion fluctuation. Further, brain cells are unable to swell due to the rigidity of the skull [19].

Hepatic encephalopathy (HE) is a significant cause of morbidity and mortality in advanced cirrhotic patients [58]. Although the mechanisms by which carnitine provides neurological protection are unknown, a systematic review of the literature confirmed that L-acyl-carnitine is promising as a safe and effective treatment for HE [58]. One suggested mechanism of carnitine action is its reduction of serum ammonia levels leading to improved psychometric measures [63-65]. Acetyl-L-carnitine is neuroprotective when administered at supraphysiological concentration [114]. There is much interest in its clinical application in various neural disorders such as Alzheimer's disease and painful neuropathies [114].

Neuronal ceroid lipofuscinoses (NCLs) are a group of autosomal-recessive hereditary lysosomal storage diseases caused by mutations in at least 8 genes (CLN1-CLN8) [115]. These disorders are characterized by massive accumulation of autofluorescent lysosomal storage bodies in most cells of the CNS and associated severe degeneration of the CNS [115]. There appears to be an anomalous storage of mitochondrial ATP synthase subunit c that is neither the result of mutation nor enhanced expression of the protein but rather a slower degradation of the mitochondrial ATP synthase in comparison with normal cells [116]. Acetyl-L- carnitine has been shown to be therapeutic in treatment of this disease [114]. Traina and colleagues suggest that ALC might rebalance the disorders underlying neuronal ceroid lipofuscinosis disease which are related to a disturbance in pH homeostasis. This lack of homeostasis affects acidification of vesicles transported to the lysosomal compartment for degradation [114].

Several investigators have studied the effect of acetyl-L-carnitine administration on older individuals with dementia [117]. Although the statistical evaluation of several of these "studies" were inadequate with some reports presenting only "clinical impressions," all investigators noted some improvement in cognitive function and positive effects of neuropsychological parameters in elderly patients with dementia subsequent to the administration of acetyl-L-carnitine [117].

An increasing number of studies have demonstrated the efficacy of secondary antioxidants, such as acetylcarnitine, to reduce or to block neuronal death that occurs in the pathophysiology of Alzheimer's disease. These studies have suggested that there may be mechanisms beyond antioxidant activities playing a neuroprotective role [118]. Based on the evidence that heat-shock proteins (Hsps) can exert neuroprotective effects against oxidative stress-related injury and that nutritional antioxidants are able to upregulate Hsps in neurons, use of nutritional antioxidants such as carnitine/acetyl-L-carnitine has been advocated to counteract the oxidative stress-induced brain damage in Alzheimer's disease [67,119].

Similarly, carnitine/acetyl-L-carnitine has been used in treatment of degenerative neuronal function in older Down's Syndrome patients since upon autopsy it was revealed that almost 100% of these patients over 40 years of age had symptoms of dementia [117]. However acetyl-L-carnitine was not found to benefit young men suffering Down's Syndrome [117].

### Heart disorders and supplementation in cardiovascular disease

Human skeletal and cardiac muscles contain relatively high concentrations of carnitine received from the plasma, since they are incapable of carnitine biosynthesis [1]. The heart is one of the organs most affected in carnitine-acylcarnitine carrier (CAC) deficiency [120]. By catalyzing the carnitine/acylcarnitine exchange, CAC allows the import of fatty acyl moieties into the mitochondria where they are oxidized by the β-oxidation pathway. This pathway is the major source of energy for the heart [120]. Cardiomyopathy, cardiac arrhythmia, (likely due to the accumulation of long-chain fatty acids and acylcarnitines that cannot be oxidized), cardiac insufficiency and respiratory distress arise from CAC deficiency [120]. Carnitine deficiency has been associated with heart failure [5].

The mechanism(s) underlying the effects of L-carnitine (LC) in cardiovascular diseases are not well clarified. Miguel-Carrasco *et al*. [121] demonstrated in a rat model that chronic administration of LC reduces blood pressure and attenuates the inflammatory process associated with arterial hypertension.

In opposition to the reported beneficial effects of carnitine overload, Diaz *et al*. [122] demonstrated that carnitine worsened the recovery of contractile function in transient ischemia. In addition, carnitine supplementation increased contracture of the heart shortly after reperfusion. Diaz and colleagues concluded that in conditions where it does not increase glucose oxidation, carnitine supplementation worsens both injury and recovery of contractile function after transient ischemia in perfused rat heart [122].

L-carnitine has been shown to have favorable effects in patients with severe cardiovascular disorders, such as coronary heart disease, chronic heart failure and peripheral vascular disease [123-125]. In patients with chronic heart disease, administration of L-carnitine over 12 months led to attenuation of left ventricular dilatation and prevented ventricular remodeling while reducing incidence of chronic heart failure and death [125]. In ischemia, L-carnitine reduces myocardial injury mainly through improving carbohydrate metabolism and by reducing the toxicity of high free fatty acid levels [124]. The protective effect of L-carnitine on ST-elevation myocardial infarction has been documented. Following an acute myocardial infarction prompt L-carnitine administration and subsequent maintenance therapy attenuates progressive left ventricular dilatation [126]. L-carnitine reduces early mortality but not overall risk of death or heart failure at 6 months [127]. L-carnitine supplementation also prevents ventricular enlargement and dysfunction, reduces the infarct size and cardiac biomarkers, and diminishes the total number of cardiac events including cardiac deaths and nonfatal infarction [128,129]. Xue and colleagues suggest that the beneficial effects of L-carnitine in cardiovascular disease are due to the resumption of normal oxidative metabolism and restoration of myocardial energy reserves [128,129].

Carnitine has been widely recommended as a supplement in cardiovascular disease. However, it should be noted as mentioned previously, in conditions where it does not increase glucose oxidation, carnitine supplementation worsens both injury and recovery of contractile function after transient ischemia in the perfused rat heart [122].

### Neuromuscular disease

Myopathy can be seen with biochemically defined defects in mitochondrial substrate transport or utilization, including the myopathic form of carnitine deficiency; CPT II deficiency (which most often presents with exercise intolerance and myoglobinuria and is discussed below) [130]. Patients with Duchenne dystrophy and Becker dystrophy showed lower carnitine levels in muscle biopsies than controls [131] though these levels were higher than in patients suffering primary carnitine deficiency as a result of severe muscle damage [1].

CPT II Type 1 "muscle" phenotype, which is the most frequent clinical presentation, is characterized by recurrent episodes of muscle pain, rhabdomyolysis (a potentially fatal disease that occurs suddenly and with great force destroying skeletal muscle) and myoglobinuria. This deficiency is often triggered by heavy exercise, and can also manifest as a result of exposure to cold, infection, emotional distress, and/or fasting [132].

### Drug interactions

Cyclosporin A induced nephrotoxicity has been discussed above. Valproic acid (VPA) is a broad-spectrum anti-epileptic drug [133]. It is usually well tolerated, but rare serious complications such as VPA-induced hepatotoxicity (VHT) and VPA-induced hyperammonaemic encephalopathy (VHE) may occur in some patients who receive VPA chronically [133]. It has been suggested that VHT and VHE may be promoted by carnitine deficiency, either pre-existing or deficiency induced by VPA [134]. VPA is used to treat psychiatric disorders and as such there is an association with accidental or deliberate overdose, the incidence of which is increasing [135,136]. Benefits of oral L-carnitine in relation to VPA-associated deficiency and related adverse effects have been reported [137-139]. Carnitine supplementation during VPA therapy in high-risk patients is now recommended by some, especially by pediatricians [133]. L-carnitine therapy could also be valuable in those patients who develop VPA-induced hepatotoxicity or VPA-induced hyperammonaemic encephalopathy [133].

Al-Majed and colleagues [140] found that carnitine deficiency and oxidative stress are risk factors during development of cisplatin (CDDP)-induced cardiomyopathy and that carnitine supplementation, using propionyl-l-carnitine, prevents the progression of CDDP-induced cardiotoxicity.

### Aging and bone loss

Adverse effects of aging are, in part, attributed to decreases in mitochondrial function and increases mitochondrial oxidant production [141]. L-carnitine levels in tissues have been found to decline with age [142]. Acetyl-L-carnitine (ALCA) fed to aged rats was shown to reverse age-related declines in tissue L-carnitine levels and also reversed a number of age-related changes in liver mitochondrial function; however, high doses of ALCA increased liver mitochondrial oxidant production [143]. Liu *et al*. demonstrated that memory loss in old rats is associated with brain mitochondrial decay and RNA/DNA oxidation. Partial reversal was obtained by feeding acetyl-L-carnitine and/or R-alpha -lipoic acid [144]. ALCA, together with alpha-lipoic acid, was shown to improve mitochondrial energy metabolism and decrease oxidative stress leading to improved memory in aged rats [144,145]. Several studies have reported that supplementing rats with both L-carnitine and alpha-lipoic acid halts age-related increases in reactive oxygen species (ROS), lipid peroxidation, protein carbonylation, and DNA strand breaks in heart, skeletal muscle and brain, concomitant with improvement in mitochondrial enzyme and respiratory chain activities [146-149]. In a clinical trial of Levocarnitine-treated elderly patients [150], there was significant improvement in total fat mass, total muscle mass, total cholesterol, LDL-C, HDL-C, triglycerides, apoA1, and apoB with concomitant decreases in physical and mental fatigue. These data suggest that administration of levocarnitine to healthy elderly subjects may result in reduction of total fat mass, and increase of total muscle mass, may be reduce fatigue and serum lipids.

Carnitine levels decrease with age [151]. Patano and colleagues suggest that this decrease in energy availability might compromise osteoblast activity and bone remodeling in an age-related manner [152]. It has been shown that cells of the osteoblastic lineage generate 40-80% of their energy demands through fatty acid oxidation [153]. Patano *et al*. [152] suggest that modulation of fatty acid oxidation may regulate the amount of energy available for protein synthesis in osteoblasts. Using an aging ovariectomized rat model they found supplementation of L-carnitine can influence bone density and slow the rate of bone turnover by slowing bone loss and improving bone microstructural properties through decreasing bone turnover [154]. The study reported that benefits of carnitine are comparable with other drugs of choice in terms of effectiveness in preventing BMD loss due to aging. Colluci and colleagues [150] used an *in vitro *model to suggest that carnitine supplementation in the elderly may stimulate osteoblast activity and decrease age-related bone loss.

### Dry eye and retinal disorders

Dry eye is a common disease of the ocular surface that is associated with corneal surface irregularity and blurred vision [155-158]. In artificial tear formulations, L-carnitine is considered a "compatible solute". Use of carnitine in artificial tears has demonstrated rapid and consistent improvements in signs and symptoms in patients with dry eye [159] suggesting an intrinsic homeostatic role for carnitine in the eye [160]. Recently, Pescosolido and colleagues [161] evaluated the presence of carnitine in tears of dry eye patients and suggested that the damage incurred on the ocular surface of dry eye patients may, in part, be due to a lack of carnitine in the tear film of these patients relative to the ocular surface cells and suggested use of solutions containing carnitine to reduce this damage. Increased tear osmolarity in dry eye disease has been found to stimulate production of inflammatory cytokines and matrix metalloproteinases by ocular surface epithelial cells [155]. Tears of patients with dry eye show significantly increased osmolarity, with a mean value of 343 mOsm compared with 302 mOsm in healthy controls [162]. Corrales and colleagues [155] showed that osmoprotectants such as L-carnitine reduce activation of (mitogen-activated protein) MAP kinases, the phosphorylation of which leads to an increased expression of cytokines, chemokines and matrix metalloproteases [155]. These factors mediate and control immune and inflammatory responses. Dysregulation of these factors in the eye can lead to corneal melting and scarring with deleterious consequences. Under hyperosmolar conditions, L-carnitine was found to protect against stress activation of corneal epithelial cells by reducing levels of kinase [155]. This activity of carnitine can by likened to the use of sunscreen reducing downstream effects of UV (pain, redness, edema, exfoliation, melanin production, collagen damage) by physically limiting the cellular damage/stress from UV, not by the pharmacological targeting of cytokines, receptors, etc. that otherwise bring about the painful sunburn. Peluso *et al*. [19] further suggest that decreased levels of carnitine in the eye in experimental diabetes (as reported by Pessotto *et al*. [163]) might be related to osmotic stress rather than pathological modification of the eye lens and that chronic aberration of osmotic pressure causes net loss of carnitine which can lead to triggering of cataracts.

Mitochondrial trifunctional protein (MTP) defects are disorders of mitochondrial fatty acid β-oxidation pathway of which progressive pigment chorioretinopathy is a long-term complication [164]. Chorioretinopathy emerges during early childhood as granular pigmentation of the central fundus with or without pigment clumping which may progress to chorioretinal atrophy, high myopia, posterior staphyloma and low vision [165]. Current treatment includes a low fat, high carbohydrate diet and avoidance of fasting which dramatically improves prognosis allowing long term survival. However the dietary impact is controversial [166]. Roomets *et al*. examined the expression of CPT-1 isoforms in photoreceptor cells and retinal pigment epithelial cells that are known to be affected morphologically and functionally in complete MTP deficiency and deficiency of long-chain 3-hydroxyacyl-CoA hydratase (LCHAD) [167]. They concluded that the mitochondrial fatty acid β-oxidation pathway probably plays an active metabolic role in retinal pigment epithelium and other neuroretinal cell types. They further suggest that accumulation of 3-hydroxylated intermediates of long-chain fatty acids may contribute to the pathogenesis of retinopathy in MTP deficiencies [164].

## Conclusion

Carnitine as a nutritional supplement has, since the 1960s, been promoted as beneficial in a number of disorders of human carnitine deficiency of impaired fatty acid oxidation, suggesting that nutritional or pharmacologic supplements of carnitine might be beneficial in some disorders [168]. However it should be noted that according to Stanley [168], over the past 40 years, there have been only two clear examples of disorders directly due carnitine deficiency that have provided evidence of unequivocal benefit from carnitine treatment.

Most healthy people, including vegetarians, produce and gain sufficient carnitine from their diets. Carnitine is thus considered a "conditionally essential" nutrient since individuals' requirements might exceed dietary intake during specific disease states. The increase of L-carnitine in plasma via oral administration, even up to and exceeding 2 mg, is limited, since L-carnitine has a very poor absorption and bioavailability, a very high renal clearance, and active uptake into tissues. Intravenous administration of L-carnitine might prove more effective, however where kidney function is not impaired, as more than 95% of L-carnitine filtered by glomeruli is retained and excess exogenous L-carnitine is readily excreted once the active transporters are saturated.

Despite this, in a number of disease states much work has been done regarding the effects of prophylactic levels of carnitine though some controversy and misconceptions relating to its use in general nutrition need to be addressed. Carnitine is a natural compound, free from toxicity when given in oral doses up to several grams and thus supplements are often recommended in primary and secondary deficiencies. Since carnitine is readily excreted, supplemental ingestion is well tolerated. Evidence from both rodent and human studies supports health-related benefits when used as a therapeutic agent.

## Competing interests

The authors declare that they have no competing interests.

## Authors' contributions

The authors' responsibilities were as follows--QG and JLF conceived and researched the review; JLF drafted the review; QG, MDW, JV and PAS provided critical discussion and revision of the article for intellectual content, and approved the final version of the manuscript.

All authors have read and approved the final manuscript.
